# AO distractor and manual traction reduction techniques repair in distal tibial fractures: a comparative study

**DOI:** 10.1186/s12891-022-06008-y

**Published:** 2022-12-12

**Authors:** Hao-Jun Wu, Yan-Xia He, Chen Hang, Lin Hao, Ting-Kui Lin

**Affiliations:** 1 The Orthopaedic Center, the Affiliated Hospital of Guangdong Medical University, No. 57 South Renmin Avenue, Xiashan District, Zhanjiang, 524001 China; 2 The Operation Room, the Affiliated Hospital of Guangdong Medical University, Zhanjiang, China; 3 The Party Committee Office, the Affiliated Hospital of Guangdong Medical University, Zhanjiang, China

**Keywords:** Distal tibia fractures, Minimally invasive plate fixation, AO distractor, Manual reduction

## Abstract

**Background:**

Minimally invasive plate osteosynthesis (MIPO) via percutaneous plate placement on the distal medial tibia can be performed with minimizes soft tissue injury and produces good clinical results. However, the difficulty with MIPO lies in how to achieve satisfactory fracture reduction and maintain that reduction via indirect reduction techniques to facilitate internal fixation. The purpose of this study was to compare the effects of AO distractor and manual traction reduction techniques combined with MIPO in the treatment of distal tibia fractures.

**Methods:**

Between January 2013 and December 2019, 58 patients with a distal tibia fracture were treated using MIPO. Patients were divided into two groups according to the indirect reduction method that was used: 26 patients were reduced with manual traction(group M), and 32 were reduced with an AO distractor (group A).Time until union and clinical outcomes including AOFAS ankle-rating score and ankle range of ankle motion at final follow-up were compared. Mean operative time, incision length, blood loss and postoperative complications were recorded via chart review. Radiographic results at final follow-up were assessed for tibial angulation and shortening by a blinded reader.

**Results:**

Mean operative time, incision length, and blood loss in group A were significantly lower than in group M(*p* = 0.019, 0.018 and 0.016, respectively).Radiographic evidence of bony union was seen in all cases, and mean time until union was equivalent between the two groups (*p* = 0.384).Skin irritation was noted in one case(3.1%) in group A and three cases(11.5%)in group M, but the symptoms were not severe and the plate was removed after bony union. There was no statistically significant difference in postoperative complications between the two groups(*p* = 0.461). Mean AOFAS score and range of ankle motion were equivalent between the two groups, as were varus deformity, valgus deformity, anterior angulation and posterior angulation. No patients had gross angular deformity. Mean tibial shortening was not significantly different between the two groups, and no patients had tibial shortening > 10 mm.

**Conclusion:**

Both an AO distractor and manual traction reduction techniques prior to MIPO in the treatment of distal tibial fractures permit a high fracture healing rate and satisfying functional outcomes with few wound healing complications. An AO distractor is an excellent indirect reduction method that may improve operative efficiency and reduce the risk of soft tissue injury.

## Background

Distal tibia fractures represent approximately 10–13% of all tibia fractures and are usually high-energy injuries, which are associated with severe comminution and soft tissue trauma [1]. These injuries are particularly difficult to manage due to the limited soft tissue coverage and poor vascularity of the distal tibia and the proximity of the fracture to the ankle joint. Skin necrosis, soft tissue infections, and non- or delayed bony union because of microvascular damage caused by periosteal stripping have been reported [2–4].

Although the quality of distal tibia fracture reduction using conventional open reduction and plate internal fixation (ORIF) is satisfactory, it increases the risk of soft tissue injury and severe vascular damage. The wound infection rate remained at a high level after after ORIF, especially in smokers and patients with systemic diseases such as diabetes and neuropathy [5].

It is critical to reduce soft tissue injury and the risk of skin complications. Minimally invasive plate osteosynthesis (MIPO) via percutaneous plate placement on the distal medial tibia can be performed without the need for a significant incision or dissection at the fracture site. This procedure minimizes soft tissue injury and produces good clinical results. However, the difficulty with MIPO lies in how to achieve satisfactory fracture reduction and maintain that reduction via indirect reduction techniques to facilitate internal fixation [6, 7]. Using the open indirect "biologic" osteosynthesis technique, MIPO was combined with an AO distractor [8, 9] or manual reduction and locked compression plating (LCP).To the best of our knowledge, no clinical study has compared the outcomes of AO distractor vs. manual reduction prior to MIPO.

The hypothesis of this study was that the clinical and radiographic results of AO distractor MIPO would be comparable with manual reduction MIPO in the treatment of distal tibia fractures.

## Methods

### Study design

This study was approved by our Institutional Review Board and informed consent was obtained from all patients prior to surgical treatment. Inclusion criteria for this study were age > 18 years old, patients with distal 1/3 closed tibial fractures who underwent plate and screw fixation with a MIPO technique, and a minimum follow-up of 12 months. Excluded criteria included patients who could not be operated on within the acute period (7–10 days), fractures with neurovascular injury, presence of an additional fracture of the tibia, pathologic fractures, patient under 18 years old, previous tibia surgery on the affected side such as deformity surgery, a follow-up period less than 12 months, and ipsilateral neurological deficit. Between January 2013 and December 2019, 70 patients were treated with MIPO for a distal tibia fracture at our institution and followed up for at least 1 year. Nine patients with an open fracture and three patients with a displaced intra-articular fracture were excluded. The remaining 58 patients were included in this study. All data were collected retrospectively. Patients were divided into two groups according their fracture reduction method that were used in the tibia: 26 patients were fixed with manual reduction MIPO (group M), and the other 32 were fixed with AO-distractor reduction MIPO (group A).

Fractures were classified using the AO/OTA fracture classification and compared between groups M and A. “Distal tibia” fractures (AO/OTA 43) were defined as fractures occurring within 5 cm of the distal tibial plafond within the distal one-third of tibial shaft [10].

### Surgical technique

Surgery was performed by the same surgeon (HJW) if the patient’s soft tissues were amenable to fixation. Patients were placed under general or spinal anesthesia in the supine position to permit radiation fluoroscopy. A tourniquet was used. For cases associated with a lateral malleolus fracture, that fracture was fixed first to reconstruct the correct length of the fibula.

**Fig. 1:**
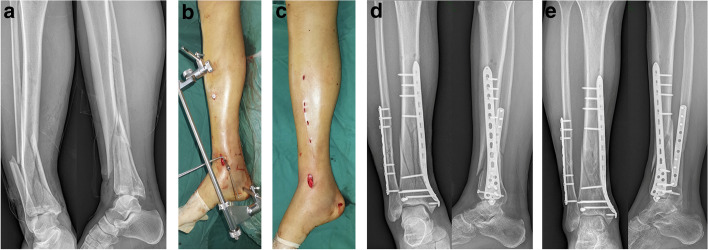
**A–E** An AO distractor was used to achieve satisfactory reduction of a distal tibia fracture. **A** Anteroposterior and lateral radiographs of a male who sustained an AO 43-C1.3 type distal tibia fracture. **B** An AO distractor was used to achieve satisfactory reduction. **C** Final view of operated limb after completion of MIPO. **D** Postoperative anteroposterior and lateral radiographs. **E** Anteroposterior and lateral radiographs 6 months postoperative showing a healed distal tibia

In group A (Fig. 1), an extension table permitted the use of an AO-Distractor (DOUBLE MEDICAL, China). A Schanz screw was inserted into the middle of the calcaneus from medial to lateral through a stab incision. A second Schanz screw was inserted into the proximal tibia through the plate parallel with the first screw in typical fashion. The distractor was assembled onto the Schanz screws, and lateral and rotational tibial deformity was corrected via manual traction. The proximal and distal fixation clips were then tightened. Distraction of the tibial to the correct length and correction of varus/valgus alignment were performed via mechanical traction on the rotating nut. Intraoperative C-arm fluoroscopy was used to evaluate the correct placement of the fractures. MIPO osteosynthesis was then performed in the usual fashion. An approximately 2–3 cm anterior curved incision was made over the medial malleolus to facilitate exposure and fixation of the anteromedial articular surface of the tibia. A subcutaneous tunnel was created along the anteromedial aspect of the tibia via blunt dissection using a large Kelly clamp. A distal tibia LCP plate (DOUBLE MEDICAL, China) was placed between the fascia and the soft tissues along the anteromedial tibial surface without stripping the periosteum or exposing the tibia. The plate was pushed percutaneously from the distal to proximal tibia, and after checking for appropriate reduction was fixed with a cancellous screw. The most proximal area of the inserted plate was palpated through the skin, and several 0.5-cm skin incisions were made along the center of the screw hole of the most proximal area of the plate. The plate was attached to the proximal tibia with a cortical screw. Fracture reduction status and plate location were then rechecked. Operative time, incision size and incision blood loss were recorded immediately after surgery. Incision size was measured only including the length of medial ankle incision and proximal tibial small incision, but not including the incision of fibula fracture. blood loss was exactly recorded by bulb suction syringes.

**Fig. 2:**
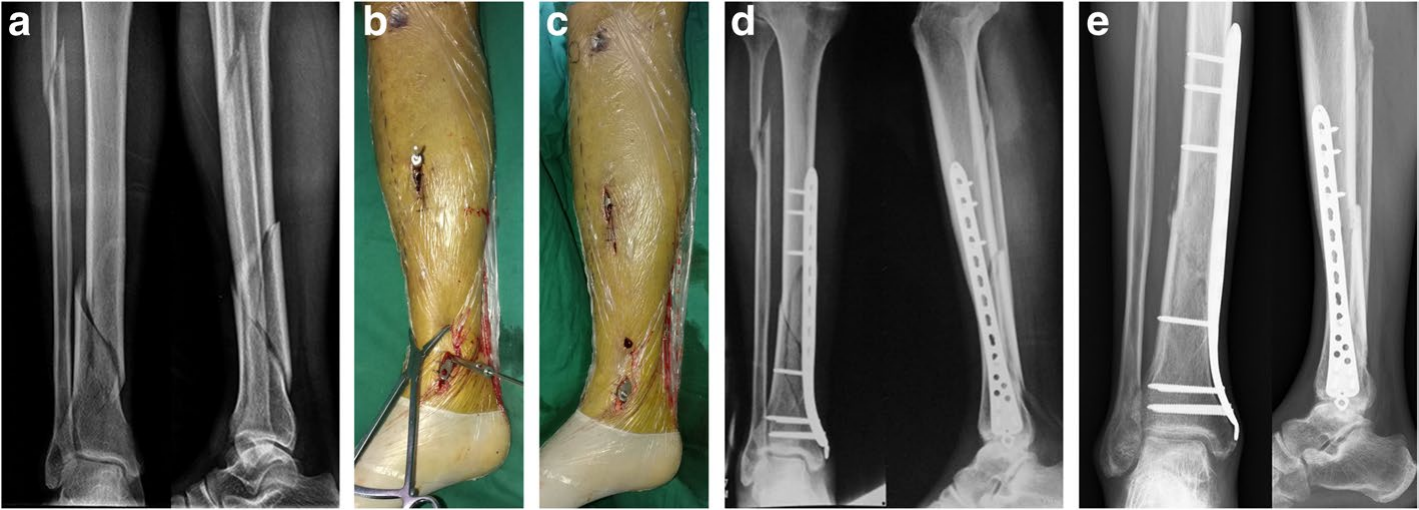
**A–E** Reduction of a distal tibia fracture using manual traction. **A** Anteroposterior and lateral radiographs of a male who sustained an AO 43-A2.3 type distal tibia fracture. **B** A manual traction reduction technique was used to achieve indirect reduction. **C** Final view of operated limb after completion of MIPO. **D** Postoperative anteroposterior and lateral radiographs. **E** Anteroposterior and lateral radiographs taken 8 months postoperative demonstrate a healed distal tibia

In group M (Fig. 2), after fixation of the fibula, indirect reduction of the distal tibial fracture was performed via manual traction under C-arm fluoroscopy. A curved medial malleolar incision through an anteromedial approach was made. Normal MIPO osteosynthesis was performed with a distal tibia medial approach [11, 12].

### Postoperative care

Patients received cefazolin for 24 h as postoperative prophylaxis [11]. Ankle and knee joint exercises were performed starting 2 days after surgery. Sutures were removed 2 weeks postoperatively and patients avoided motion exercises during that period to lessen the shear forces under the skin flap. After the soft tissues healed, partial weight bearing was allowed starting 4 to 6 weeks after surgery for all patients. Gradual weight-bearing was encouraged. Full weight-bearing was permitted when there was radiographic evidence of bony union and no pain at the fracture site.

### Postoperative assessments

Radiographic union was defined as the appearance of a mature callus on 3 or 4 planes, and clinical union as painless full weight-bearing. The soft tissues were evaluated and wound infections, necrosis, tenderness due to the plate and plate removal were recorded. Clinical results were assessed using the AOFAS ankle-rating system [13], and ankle range of motion at final follow-up was compared between groups. Mean operative time and postoperative complications were identified on a retrospective chart review.

Radiographic evaluations were performed at regular intervals: 6 weeks, 3 months, and monthly until radiographic healing was confirmed. Radiographs were assessed by measuring tibial angulation and shortening at final follow-up. Angulation was measured using the method previously reported by Milner [14]. Shortening was evaluated via comparison with the unaffected side. To avoid potential bias, an independent observer who was not part of the operative team assessed the radiographs.

### Statistical analysis

Data are presented as mean, standard deviation, frequency, median, lowest and highest value, and ratio as appropriate. Normality was measured using the Kolmogorov–Smirnov test. The Mann–Whitney test was used for quantitative comparisons. The Chi-Square test was used to compare qualitative data, and Fischer’s Exact test was used when Chi-Square test conditions were not met. Data were analyzed using SPSS 19.0 (IBM, Armonk, NY, USA). Statistical significance was defined as *p* < 0.05.

## Results

A total of 58 patients were evaluated. In group M, mean patient age was 43 (19–63) years, seventeen of the 26 patients were male and mean follow-up was18.5 (12–25) months. In group A, mean patient age was 44.3 (18–68) years, 21 of the 32 patients were males and mean follow-up was 17.3 (14–20) months. The demographics of the two groups are listed in Table 1. Eleven cases in group M and fifteen cases group A had an associated fibular fracture. No difference was found between the two groups in terms of age, sex, injury mechanism, fracture pattern, associated fibular fracture, time until surgery and post-operative follow-up.

**Table 1:** Patient Demographics

	Group M	Group A	*P* value
No. of patients	26	32	
Sex (male/female)	9/17	11/21	0.796
Age (years)	43(19–63)	44(18–68)	0.809
Injury mechanism			0.837
Traffic accident	14(53.8%)	17(53.1%)	
Slip down	11(42.3%)	12(37.5%)	
Fall from a height	1 (3.8%)	3 (9.4%)	
Fracture pattern			0.796
43A1	8 (30.7%)	10(31.3%)	
43A2	7 (26.9%)	8 (25%)	
43A3	4 (15.4%)	3 (9.4%)	
43B1	4 (15.4%)	5 (15.6%)	
43B2	1 (3.8%)	2 (6.3%)	
43B3	0	1 (3.1%)	
43C1	2 (7.7%)	2 (6.3%)	
43C2	0	1 (3.1%)	
Associated fibular fracture	11(42.3%)	15(46.9%)	0.934
Time before surgery (days)	5.55 ± 2.53	5.96 ± 2.29	0.553
Follow-up (months)	18.52 ± 6.58	17.283 ± 2.53	0.694

The mean operative time of 67.24 ± 18.69(range, 40-110 min), incision length of 5.47 ± 1.39 (range, 3-7 cm) and estimated blood loss of 16.03 ± 11.98(range, 5–50 ml) in group A were significantly lower than those of group M (operative time 82.39 ± 26.45 (range, 50-130 min), incision length 6.42 ± 1.58 (range, 3-9 cm) and estimated blood loss26.52 ± 18.18 (range, 10-60 ml)), *P*-values were 0.019, 0.018 and 0.016, respectively (Table 2).

**Table 2:** Comparison of the clinical results of group M and group A Operative time (min)

	Operative time (min)	Incision length (cm)	Blood loss (ml)	Complications	Time until fracture union(months)
Group M	82.39±26.45	6.42±1.58	6.42±1.58	3(11.5%)	7.09±1.62
Group A	67.24±18.69	5.47±1.39	16.03±11.98	1(3.1%)	6.72±1.36
*P* value	0.019	0.018	0.016	0.461	0.384

Radiographic evidence of bony union was seen in all cases. Mean time until union was 6.72 ± 1.36 (range, 4–8 months) in group A and 7.09 ± 1.62 (range, 4–9 months) in group M (*p* = 0.384, Table 2). Skin irritation was observed in one case(3.1%) in group A and three cases(11.5%)in group M, but symptoms were not severe and the plate was removed after bony union. There were no statistically significant differences in postoperative complications between the two groups(*p* = 0.461, Table 2).

At final follow-up, the median AOFAS score in group A(90) was significantly higher than that of group M (88, *P* = 0.211).Mean dorsiflexion of 37.91 ± 2.89 (range, 0–50°) and plantarflexion of 14.34 ± 2.01 (range, 0–20°) in group A were slightly better than the mean dorsiflexion of 36.77 ± 3.44° and plantarflexion of 13.54 ± 1.98° in group M, These were not statistically significant (*p* = 0.177 and 0.133, respectively, Table 3).

**Table 3:** Comparison of the functional results of group M and group A

	AOFAS Score	Range of motion(°)	
		Dorsiflexion	Plantarflexion
GroupM	88.85 ± 4.07	36.77 ± 3.44	13.54 ± 1.98
Group A	90.19 ± 3.98	36.77 ± 3.44	14.34 ± 2.01
*P* value	0.211	0.177	0.133

There were no significant differences in mean varus deformity, valgus deformity, anterior angulation or posterior angulation between the two groups (Table 4). No patients developed an angular deformity. Mean tibial shortening was not significantly different between the two groups, and no patient shortened > 10 mm.

**Table 4:** Comparison of the radiographic results for group M and group A Varus deformity (°)

	Varus deformity (°)	Valgus deformity (°)	Anterior angulation(°)	Posterior angulation (°)	Shortening (mm)
Group M	2.15 ± 1.19	2.58 ± 1.24	2.85 ± 1.59	3.08 ± 1.87	3.54 ± 2.25
Group A	1.94 ± 0.84	2.06 ± 0.91	2.34 ± 1.07	2.31 ± 1.09	2.81 ± 1.45
*P* value	0.421	0.074	0.157	0.057	0.142

## Discussion

Fractures of the distal tibia are usually the result of combined compressive and shear forces and may involve metaphyseal instability, articular depression and soft tissue injury. Injury complexity, lack of muscle coverage and poor vascularity make these fractures difficult to treat. The ideal treatment modality for distal tibial fractures is controversial, and depends on fracture morphology, displacement and comminution. Surgical treatment of distal tibial fractures includes several options: external fixation, intramedullary (IM) nailing, ORIF and MIPO. However, none of these methods are ideal. External fixation is very useful as a temporary option for skeletal and soft tissue traction, but may result in malalignment, malunion, nonunion, pin tract infection and ankle stiffness if used as a definitive treatment method [15, 16]. IM nailing has been proposed as a good option due to the biologic concepts of fixation and load sharing. However, concerns about the routine use of IM nails for distal tibial fractures include instability, malunion and non-union [17]. These risks are particularly concerning in the setting of an open fracture, where a higher incidence of complications was noted after IM nailing. IM nailing was also associated with greater post-operative malalignment than plating [18, 19]. Precise reduction of the articular fragments can be achieved with ORIF, and this methodis therefore used to restore the anatomic structures of the joint surface. However, ORIF requires widespread dissection of the soft tissues, which can lead to increased rates of infection and soft tissue complications. ORIF has also been found to alter the blood supply of the tibia, which may lead to delayed union or nonunion [2–4].

The MIPO technique permits indirect fracture reduction and stable fixation with minimal soft tissue dissection by maintaining the fracture hematoma. The fracture is primarily reduced by ligamentotaxis, and plating is performed percutaneously using a minimally invasive technique. The use of indirect reduction techniques has led to improved fracture-healing and reduced the need for bone-grafting compared with older direct reduction techniques [20].Consequently, MIPO has recently been recognized as an ideal choice for the treatment of distal tibial fractures [6–9, 21].

To maximize the advantages of MIPO osteosynthesis over other fracture reduction techniques, exposure of the metaphyseal and diaphyseal fragments should be avoided. This means that careful indirect reduction of the fracture is necessary prior to definitive fixation. Not only is indirect reduction a challenge for surgeons who are used to open techniques, but the maintenance of any achieved reduction can be difficult until it is held in place with the plate. Indirect fracture reduction often necessitates applying considerable force to displace one or both ends of the broken bone against the natural forces of the muscles and tendons.

Manual traction is an indirect reduction technique. Manual traction is performed by the surgeon and medical staff, occasionally with the help of an image intensifier to evaluate the reduction. There is always danger of over-distraction with this technique, which may result in unnecessary muscle, tissue and ligament strain during the manipulation process, and of excessive x-ray exposure that is undesirable for both the patient and the medical staff. Furthermore, due to the large holding forces that are necessary to reduce the fracture, exact ‘first time’ positioning is virtually impossible. What is needed is a mechanical system to permit the precise positioning of the fractured bone ends without the need for multiple docking attempts. Bony fragments need to be held in correct alignment as long as necessary while osteosynthesis is performed [22].

An AO distractor [8, 9] is a device-assisted indirect reduction technique. After Schanz screws are placed into the proximal and distal parts of the fracture, the fracture is distracted and reduced by manipulating the mobile elements of the device (Fig. 1B). Our study demonstrates that the use of an AO distractor results in a significantly shorter operative times, smaller incision lengths and lower blood loss compared with manual reduction alone. These differences indicate that an AO distractor is a satisfactory reduction device that can improve the efficiency of the operation. The reason for the benefits of the AO distractor may be that it helps to reduce the strain and fatigue that often accompanies manual traction. This percutaneous technique minimizes soft tissue insult and the disruption of fracture biology. Moed et al. [23] presented a series of 44 fractures of the tibia that required operative stabilization and were treated using an intra-operative external transfixion pin frame and IM nailing. For added reduction stability, the carbon fiber rod on the concave side of the angular deformity can be replaced with an AO/ASIF universal distractor. The authors’ technique shortens setup time, provides complete access to the distal tibia, and permits free manipulation of the limb, thereby facilitating nail insertion and placement of distal locking screws.

To the best of our knowledge, no clinical study has previously compared the outcomes of AO distractor-and manual traction-mediated MIPO for distal tibial fractures. The present work found no significant differences between the two techniques in terms of time until union, AOFAS score and range of motion. Kim et al. [24]reported excellent AOFAS scores after MIPO and minimal ORIF (86.0 and 86.7, respectively) in patients with distal tibial fractures. Our study found that the mean AOFAS ankle scores were 90 in group A and 88 in group M, which were not significantly different. The satisfactory results following both treatment methods were probably because most of our cases were AO/OTA type A or type B distal tibia fractures (Table 1). Prior use of MIPO on AO/OTA type C distal tibia fractures was associated with worse functional outcomes compared with type A and B fractures. The complexity of AO/OTA type C fractures is mainly due to their commonly high-energy injury mechanisms, which have been shown to have higher complication rates and poorer long-term results than lower-energy partial articular type B or extra-articular type A fractures [25].

Complications associated with the use of MIPO in distal tibial fractures include skin impingement, malunion, delayed union and intra-operative saphenous nerve and vein injury [26, 27]. One patient (3.1%) in group A and three (11.5%) in group M that developed skin irritation, which was characterized by discomfort and pain around the medial malleolus. However, these symptoms were not severe and the plate was removed after bony union. No statistically significant differences in postoperative complications were observed between the two groups. Medial plates can cause skin irritation because they are located directly under the skin and the subcutaneous soft tissues on the medial side of the distal tibia are thin. Plate exposure can also occur when the medial plate is inserted [28]. The skin incisions used in the present work were limited to one 2-cm-long anterior curved incision for plate entry and several 0.5-cm-long stab incisions for locking screws (Fig. 1C). Indirect reduction and subcutaneously applied plates respect the soft tissues and the periosteal blood supply. AO distractor and manual traction-mediated reduction of distal tibia fractures can not only improve operative efficiency, but also significantly reduce soft tissue injury.

Radiographic results were assessed at the final follow-up. There were no significant differences in mean varus deformity, valgus deformity, anterior angulation or posterior angulation between the groups. No patients had gross angular deformity. Mean tibial shortening was not significantly different between the groups, and no patient had tibial shortening of > 10 mm. Hasenboehler et al. [29] showed that varus or valgus angulation exceeding 5°, recurvatum or procurvatum exceeding 5° or rotation and shortening is beyond 5° represents significant malalignment. Sagittal plan deformity remains a common complication of MIPO. Malalignments of up to 20° are frequently observed. Vallier et al. [18] showed that 55% of patients who went onto malunion had open fractures, and that 85% of patients with malalignment after IM nailing did not require fibula fixation. The lack of adequate reduction aids explains the significant incidence of post-traumatic malalignment of the lower extremity. Our study demonstrates that the use of an AO distractor can significantly lower the rate of coronal plane malalignment compared with manual reduction alone, although there was no significant difference between the two groups. Malalignment may also be minimized by reducing the fracture via manual traction, maintaining that reduction with an AO reduction device and then fixing the fibula.

A limitation of this study was that no smoking and diabetes patients were included, although studies have shown that these conditions can lead to increased wound complications [3]. There were also few subjects in each group.

## Conclusion

AO distractor and manual traction MIPO for treating distal tibial fractures both have a high rate of fracture healing and a satisfactory functional outcomes with few wound healing complications. These two procedures are therefore both reasonable options for treating distal tibia fractures. AO distracters are an excellent indirect reduction device that can improve operative efficiency and reduce the risk of soft tissue injury. However, further long-term studies that include a large sample size are required before any definitive conclusions can be made.

## Data Availability

The datasets used and/or analyzed during the current study are available from the corresponding author on reasonable request.

## Abbreviations

MIPO: Minimally invasive plate osteosynthesis
AO: Arbeitsgemeinschaftfür Osteosynthesefragen
OTA: Orthopaedic trauma association
ASIF: Association for the study of internal fixation
AOFAS: American orthopaedic foot and ankle society
ORIF: Open reduction and plate internal fixation
IM: Intramedullary
